# Oligosaccharides from Palm Kernel Cake Enhances Adherence Inhibition and Intracellular Clearance of *Salmonella enterica* Serovar Enteritidis In Vitro

**DOI:** 10.3390/microorganisms8020255

**Published:** 2020-02-14

**Authors:** Rui Qing Foo, Mohammad Faseleh Jahromi, Wei Li Chen, Syahida Ahmad, Kok Song Lai, Zulkifli Idrus, Juan Boo Liang

**Affiliations:** 1 Institute of Tropical Agriculture and Food Security, Universiti Putra Malaysia, Serdang 43400 UPM, Selangor, Malaysia; fooruiqing@gmail.com (R.Q.F.); mfjahromi@gmail.com (M.F.J.); Wlchen.jess@gmail.com (W.L.C.); zulidrus@upm.edu.my (Z.I.); 2 Arianabiotech co. No 118, Parsian Industrial Zone, Mashad 9354195366, Khorasan Razavi, Iran; 3 Faculty of Biotechnology and Biomolecular Sciences, Universiti Putra Malaysia, Serdang 43400 UPM, Selangor, Malaysia; syahida@upm.edu.my; 4 Health Sciences Division, Abu Dhabi Women’s College, Higher Colleges of Technology, Abu Dhabi 41012, UAE; lkoksong@hct.ac.ae; 5 Office of the Deputy Vice Chancellor (Research & Innovation), Universiti Putra Malaysia, Serdang 43400 UPM, Selangor, Malaysia

**Keywords:** palm kernel cake, fructooligosaccharide, mannanoligosaccharide, *Salmonella* Enteritidis, lactate dehydrogenase, non-digestible oligosaccharides, prebiotics

## Abstract

*Salmonella enterica* serovar (ser.) Enteritidis (*S.* Enteritidis) is a foodborne pathogen often associated with contaminated poultry products. This study evaluated the anti-adherence and intracellular clearance capability of oligosaccharides extracted from palm kernel cake (PKC), a by-product of the palm oil industry, and compared its efficacy with commercial prebiotics— fructooligosaccharide (FOS) and mannanoligosaccharide (MOS)—against *S.* Enteritidis in vitro. Based on the degree of polymerization (DP), PKC oligosaccharides were further divided into ‘Small’ (DP ≤ 6) and ‘Big’ (DP > 6) fractions. Results showed that the Small and Big PKC fractions were able to reduce (*p* < 0.05) *S.* Enteritidis adherence to Cancer coli-2 (Caco-2) cells at 0.1 mg/ mL while MOS and FOS showed significant reduction at 1.0 mg/mL and 10.0 mg/mL, respectively. In terms of *S.* Enteritidis clearance, oligosaccharide-treated macrophages showed better *S.* Enteritidis clearance over time at 50 µg/mL for Small, Big and MOS, while FOS required a concentration of 500 µg/mL for a similar effect. This data highlights that oligosaccharides from PKC, particularly those of lower DP, were more effective than MOS and FOS at reducing *S.* Enteritidis adherence and enhancing *S.* Enteritidis clearance in a cell culture model.

## 1. Introduction

The human gastrointestinal tract is home to a vast number of microbes, with the large intestine containing up to 10^11^ bacterial cells per gram of colonic content [1,2]. Most of these microbes are anaerobic in nature and exist as commensals, i.e., they do not cause harm and may even be beneficial to the host in a stable and healthy environment [3].

However, the composition of the gut microbiota may change depending on the type of microbes ingested [4]. For example, the consumption of probiotics may improve gut health by increasing the number and diversity of beneficial microbes while decreasing the population of harmful pathogens [4]. Alternatively, dysbiosis, i.e., the disruption of balance within the gut microbiota due to antibiotics, diets and/or infections may lead to an overgrowth of pathogenic microbes [5,6,7].

*Salmonella*, particularly those of the serovar Enteritidis, is an example of a commonly encountered foodborne pathogen that is often associated with the consumption of contaminated poultry meat and eggs [8]. These pathogens exist as a zoonotic microbe that can be transmitted from animals to humans. This does present a challenge, as poultry meat is widely consumed in many countries. For example, in the European Union (EU), it is reported that 31.3% of *S.* Enteritidis outbreaks were associated with eggs and egg products, while in countries such as China, Vietnam and Malaysia, the presence of *S.* Enteritidis in retail chicken meat ranged from 1.1% to 20.2% of the samples collected [9,10,11,12].

*Salmonella* infection may be classified into typhoidal and non-typhoidal [13]. Typhoidal infections are caused by *Salmonella enterica* serovar (ser.) Typhi, Sendai, and Paratyphi A, B, or C [13]. These serovars are highly adapted to humans as hosts and are the cause of the deadly typhoid (caused by *S.* Typhi) and paratyphoid fever (caused by *S*. Paratyphi) [13]. Non-typhoidal *Salmonella* infections (NTS) on the other hand, are caused by *Salmonella* serovars with a broad host range such as *S.* Enteritidis and *Salmonella enterica* serovar (ser.) Typhimurium (*S.* Typhimurium) [13]. NTS usually starts with the ingestion of contaminated food [5]. Once inside the gastrointestinal tract, the bacterium induces intestinal inflammation, which leads to the formation of tetrathionate [14,15]. Under anaerobic conditions, such as that present in the gastrointestinal tract, *Salmonella* is able to utilize tetrathionate as an electron acceptor and metabolize ethanolamine; another metabolite released from damaged epithelial cells [14,15]. The utilization of ethanolamine as a carbon source in the presence of tetrathionate is a feat that not many gut microbes possess and this gives *Salmonella* a competitive growth advantage which leads to a higher rate of colonization and expansion within the gut [14,15]. Usually NTS infections are self-limiting but in immunocompromised individuals, complications may occur [14]. Such complications are often typhoid-like in nature and these invasive strains of NTS are termed invasive non-typhoidal *Salmonella* infections (iNTS) [16]. In iNTS, the pathogen takes advantage of and invades the microfold cells located in the Peyer’s Patches [5,16]. Alternatively, *Salmonella* may also directly breach the epithelial barrier of the gastrointestinal tract by dysregulating the tight junction proteins of the epithelial cells [16]. Once the pathogen has breached the intestinal epithelial barrier and entered the lamina propria, the microbe then encounters the innate branch of the immune system. Depending on the *Salmonella’*s serovar and virulency, the pathogen may invade and replicate within the host’s macrophages [16]. If left unchecked, this may lead to a systemic *Salmonella* infection [16]. Current methods of stemming the spread of salmonellosis involves *Salmonella* control programs in poultry populations and food preparation compliance [12].

There are findings suggesting that the inclusion of non-digestible oligosaccharides (NDOs) in one’s diet may play a protective role against some *Salmonella enterica* serovars. For example, NDOs have been reported to inhibit the adhesion of *S.* Fyris to Cancer coli-2 (Caco-2) cells, improve *S.* Enteritidis clearance in macrophages and reduce colonization of *S.* Enteritidis in broiler chicks [17,18,19]. If the inclusion of a diet supplemented with NDOs may improve food quality and safety through the prevention of pathogen colonization and the improvement of animal and consumer health, it appears to be an idea worth pursuing [20].

Common sources of NDOs are often plant or microbial based. For example, fructooligosaccharide (FOS) and inulin are commercially produced from chicory roots, while oligosaccharides such as mannanoligosaccharide (MOS) are obtained from yeast cell walls [21,22].

However, the growing of crops solely for the purpose of feed supplementation may not sit well in the era of limited land resources and sustainability and the extraction of MOS from yeast involves costly enzymatic degradation of its polysaccharide to yield mannan oligosaccharide and mannose [23,24,25]. It would be ideal if multiple uses of a crop and its by-products could be utilized prior to disposal in order to maximize its utility and commercial value. Currently, palm kernel cake (PKC) has been successfully incorporated as a cost-saving feed ingredient for ruminants [26]. However, Jahromi et al. (2016) has reported that PKC may be a source of high value NDOs [27].

While the purported protective benefits of these oligosaccharides appear to be promising, different types and sizes of oligosaccharides behave differently when administered. For example Ito et al. (2011) reports that fructans (a polymer of fructose) with a lower degree of polymerization (DP) was better at increasing immunoglobulin A (IgA) production in rats, while Biggs et al. (2007) reports that short-chain fructooligosaccharide and MOS were able to reduce the pathogen *Clostridium perfringens* in chicks but not inulin, oligofructose or transgalactooligosaccharide [28,29]. In light of the above knowledge and based on the mode of *Salmonella* infection starting from the epithelial cells of the intestinal lining to the macrophages of the lamina propria, this study evaluated the protective effects of different sized oligosaccharides extracted from PKC against *Salmonella* Enteritidis in vitro and compared their efficacy with two commercial prebiotics—FOS and MOS.

## 2. Materials and Methods

### 2.1. Sample Preparation

Palm kernel cake was purchased from a local palm kernel oil mill and oligosaccharides from PKC were obtained based on the method of Jahromi et al. (2016) with some modifications [27]. Briefly, 200 g of PKC was placed in a 1L Schott bottle and topped up with distilled water to 1L. Then, the mixture was autoclaved at 121 °C for 20 min. After autoclaving the aqueous extract was filtered using a Whatman filter paper no.1 (GE Healthcare Life Sciences, Marlborough, MA, USA). After filtration, the aqueous extract was concentrated under reduced pressure to approximately half its initial volume using a rotary vacuum evaporator (Heidolph Instruments GmbH & Co. KG, Schwabach, Germany). The rotary vacuum evaporator was operated at 100 mbar, 60 °C and 100 rpm. Liquid–liquid extraction was then performed using aqueous extract: chloroform (50:50, v:v). The aqueous layer on the top was collected and the chloroform layer at the bottom was discarded. The liquid–liquid extraction was repeated for a second time. The aqueous layer was collected and centrifuged at 3000 rpm for 10 min and the precipitate was discarded. Liquid–liquid extraction was repeated for the third and final time. The aqueous layer was collected and concentrated once more to approximately half its volume. To avoid damaging the High Performance Liquid Chromatography (HPLC) column in subsequent downstream analysis, acetonitrile was then added to the aqueous extract (50:50, v:v) to precipitate out compounds which are insoluble in the 50% acetonitrile solution. The acetonitrile: aqueous extract mixture was then centrifuged at 3000 rpm for 10 min and the precipitate was discarded. The remaining aqueous extract was then concentrated under reduced pressure using a rotary vacuum evaporator to remove as much water as possible before being freeze dried (FreeZone 6 Liter Benchtop, Labconco Corporation, Kansas City, MO, USA) and stored in a −80 °C freezer.

### 2.2. Size Exclusion Chromatography

In order to separate the PKC oligosaccharides based on size, 1.0 g of freeze-dried extract was dissolved in 5 mL of ultrapure water. The reconstituted extract was then filtered through a 0.22 micron syringe filter before being fed into a chromatography column (2.5 × 100 cm) packed with Bio-gel P-2, Fine gel (Bio-Rad Laboratories, Hercules, CA, USA). Using a perista pump (Atto Corporation, SJ-1211II-H, Tokyo, Japan), sample elution was carried out using degassed ultrapure water at a flowrate of 1.1 mL/min. Fractions were collected using Waters fraction collector III (Waters Corporation, Milford, MA, USA). The quantity of each fraction collected per run was 2 mL. Each fraction was then freeze dried and stored in a −80 °C freezer until required.

### 2.3. Pooling Fractions Based on HPLC Profiles

Freeze-dried fractions were dissolved in ultrapure water to a concentration of 5 mg/mL and filtered through a 0.22 micron syringe filter prior to analysis using HPLC (Waters 2690, Waters Corporation, Milford, MA, USA). The conditions for running the HPLC analysis were based on the parameters employed by Jahromi et al. (2016). Oligosaccharides in the collected fractions were detected using a reflective index (RI) detector (Waters 2414, Waters Corporation, Milford, MA, USA). The detector temperature was set at 30 °C and sensitivity was set at 1024. The column used for HPLC analysis was a 250 × 4.6 mm COSMOSIL Sugar-D column (Nacalai Tesque Inc., Kyoto, Japan) with an internal diameter of 5 µm. The column temperature was set at 35 °C and the flowrate at 0.8 mL/min. In order to group the PKC oligosaccharides based on their degree of polymerization, five oligosaccharide standards ranging from a degree of polymerization (DP) of two to six were used. These standards were mannobiose, mannotriose, mannotetraose, mannopentaose, and mannohexaose (Megazyme, Wicklow, Ireland) as Jahromi et al. (2016) reports that mannose is the main constituent of PKC oligosaccharides [27]. Once the HPLC profiles of all fractions were obtained, the fractions were pooled into two groups. The first group (Big) contained oligosaccharides with DP more than six (DP > 6) and the second group (Small) contained oligosaccharides with DP less than or equal to six (DP ≤ 6). These two pooled fractions were then freeze dried and stored in a −80 °C freezer until required.

In addition to the Big and Small PKC oligosaccharide fractions, two other commercial oligosaccharides MOS (Henan Junda Biological Technology Co., Ltd., Henan, China) and FOS (Quantum Hi-Tech (China) Biological Co., Ltd., Guangdong, China) were used in this study.

### 2.4. Molecular Weight Determination

To determine the molecular weights of the PKC oligosaccharide fractions as well as that of the commercial MOS and FOS, the samples were dissolved in distilled water, filtered through a 0.22 micron syringe filter and diluted to 10 ppm prior to being sent to Agro-Biotechnology Institute, Malaysia for targeted as well as non-targeted analysis using Liquid Chromatography Quadrupole Time-of-Flight Mass Spectrometry (LC-QTOF/MS) (6550 Series, Agilent Technologies, Santa Clara, CA, USA). The liquid chromatography conditions are as follows: (i) the mobile phase consisted of 65% acetonitrile in water; (ii) the column temperature was set at 35 °C; (iii) the injection volume was 3.00 µl and; (iv) the flow rate was set at 0.30 mL/min. The column used for the LC is a Thermo Hypercarb 3 micron × 2.1 mm × 150 mm column (Thermo Fisher Scientific, Waltham, MA, USA). The LC-QTOF/MS utilizes a dual Agilent Jet Stream electrospray ionization (ESI) as its ion source and the samples were run in the negative mode. The parameters of the ESI were as follows: (i) gas temperature at 290 °C; (ii) gas flow at 11 L/min; (iii) nebulizer at 40 psig; (iv) sheath gas temperature at 320 °C and; (v) sheath gas flow at 11 L/min. Data was obtained within the range of 100 to 1700 m/z. The oligosaccharide standards used are similar to that listed for the HPLC analysis while the monosaccharide standards consisted of a mixture of xylose, fructose, mannose, glucose and galactose (Sigma-Aldrich, St. Louis, MO, USA). For oligosaccharides larger than six DP, it is possible to calculate the expected molecular weight of these oligosaccharides as each additional sugar monomer (C_6_H_10_O_5_) increases the expected molecular weight by 162.14 atomic mass units (amu). Once the expected molecular weight is known, a screening of the oligosaccharide samples for compounds with corresponding molecular weights could be carried out using LC-QTOF/MS. Decasaccharide was chosen as the cutoff point as oligosaccharides are typically defined as low molecular weight polymers of sugar consisting of three to ten units of monosaccharides [30].

### 2.5. Cells and Bacterial Culture Conditions

Both the Caco-2 and human histiocytic lymphoma cell line(U-937) were purchased from American Type Culture Collection (ATCC). Caco-2 cells were routinely maintained in minimum essential media (MEM) containing Earle’s salts, L-glutamine and non-essential amino acids (Nacalai Tesque, Kyoto, Japan), supplemented with 10% (v:v) fetal bovine serum (HexCell Berlin GmbH, Germany) and 1% (v:v) penicillin- streptomycin solution (Nacalai Tesque, Kyoto, Japan) with a final concentration of 100 Units/mL and 100 µg/mL for penicillin and streptomycin, respectively. The temperature and atmosphere of the incubator was maintained at 37 °C and 5% CO2, respectively.

U-937 cells were grown in Roswell Park Memorial Institute (RPMI) 1640 medium containing L-glutamine (Nacalai Tesque, Kyoto, Japan) and supplemented with 10% (*v*/*v*) fetal bovine serum and 1% (*v*/*v*) penicillin- streptomycin solution, respectively. Similar to the Caco-2 cells, the temperature and atmosphere of the incubator for U-937 was maintained at 37 °C and 5% CO2, respectively.

*S.* Enteritidis was obtained from Veterinary Research Institute (VRI, Ipoh, Malaysia), Ipoh, Malaysia and was maintained as glycerol stocks in a −80 °C freezer. When required, the glycerol stocks were thawed, and the bacteria cells were resuscitated in nutrient broth (Difco) overnight at 37 °C. After an overnight incubation at 37 °C, the bacterial cells were centrifuged at 3220 rcf for 10 min. The supernatant was discarded, and the bacterial cells were resuspended in phosphate buffered saline (PBS) for cell counting. Subsequent dilutions for standardization were made with MEM medium.

### 2.6. Adherence Inhibition Study Using Caco-2 Cells

The anti-adhesion properties of oligosaccharides against *S.* Enteritidis were studied using the methods published by Coppa et al. (2006) and Ibuki et al. (2011) with some modifications [17,18]. Caco-2 cells were seeded in a 24-well tissue culture plate at a density of 1 × 10^5^ cells per well. In order to achieve post confluence and allow for enterocytic differentiation, the cells were maintained for 21 days. After 21 days, the cells were washed thrice with serum-free and antibiotic-free MEM media. Then, 0.25 mL of antibiotic-free growth medium containing either Big, Small, FOS or MOS oligosaccharides at concentrations of 0.1, 1.0 and 10.0 mg/mL were added to the cells and the cells with their respected oligosaccharide treatments were incubated for two hours. For the control, only 0.25 mL of antibiotic-free growth medium was added to the cells prior to the two-hour incubation. After two hours, 0.25 mL of MEM medium containing *S.* Enteritidis (approximately 2.8 × 10^8^ CFU/mL) was added to all wells and the cells were incubated at 37 °C for another 30 min to allow for *S.* Enteritidis adherence. The cells were then rinsed thrice with serum-free and antibiotic-free MEM media. After rinsing, the cells were lysed for 5 min with 0.5 mL of chilled PBS containing 0.1% Triton X-100 (Nacalai Tesque, Japan). Once lysed, the lysate was serially diluted and plated onto brilliant green agar (BGA) (Difco). The agar plates were then incubated overnight at 37 °C in order to enumerate the adhered *S.* Enteritidis. The percentage inhibition of *S.* Enteritidis was calculated using the equation:

(1) $$\mathrm{Percentage}\;(\%)\;\mathrm{inhibition}\;=\;(\frac{A-B}{A})\;\times \;100$$

where ‘A’ is the number of *S.* Enteritidis (CFU/mL) found on untreated (control) cells and ‘B’ is the number of *S.* Enteritidis (CFU/mL) found on cells treated with oligosaccharides.

### 2.7. LDH Analysis of Caco-2 Cells

Caco-2 cells were grown and maintained for 21 days in a 24-well plate at a seeding density of 1 × 10^5^ cells per well. Then, the cells were washed thrice with serum-free and antibiotic-free MEM media before growth medium containing 40 ηg/mL lipopolysaccharide (LPS) and either Big, Small, FOS or MOS oligosaccharides were added to the individual wells. The concentrations of oligosaccharides used were based on the results obtained from the Caco-2 adhesion inhibition study. For the negative control, only growth media was used while only LPS from *S.* Enteritidis at a concentration of 40 ηg/mL was used as the positive control to induce cellular damage. The cells in their respective treatments were incubated for 24 h. After 24 h, the supernatant from the wells were collected, centrifuged 3220 rcf for 10 min to remove cellular debris and stored in a −80 °C freezer for lactate dehydrogenase (LDH) analysis. The LDH assay was carried out based on the instructions provided by the CytoSelect LDH Cytotoxicity Assay kit (Cell BioLabs Inc., San Diego, CA, USA). Briefly, 90 µl of the collected cell culture supernatant was placed into 96-well plates. Then, 10 µl of the LDH cytotoxicity assay reagent was added to the cell culture supernatant. The plates were then incubated at 37 °C for 30 min before the absorbance at 450 nm was read using a microplate reader (RT-2100C Rayto, Rayto Life and Analytical Sciences Co., Ltd., Shenzhen, China). Three biological replicates were performed for the LDH analysis. Each biological replicate consists of cell culture supernatants pooled from three different wells of a 24-well plate and each pool has two technical replicates for the microplate readout.

### 2.8. Intracellular Salmonella Clearance Using U-937

The intracellular *S.* Enteritidis clearance was carried out according to the method employed by Ibuki et al. (2011) with some modifications [18]. U-937 monocytes were suspended in growth medium containing 100 ηg/mL phorbol 12-myristate 13-acetate (PMA) (Sigma) and were seeded in a 24-well tissue culture plate at a density of 2 × 10^5^ cells per well. These PMA-treated cells were incubated for three days before being washed thrice with RPMI. The cells were then incubated for an additional five days in PMA free medium. Once differentiated into macrophages, the U-937 cells were washed thrice with serum-free and antibiotic-free RPMI media. Then, 0.25 mL of antibiotic-free growth medium containing either Big, Small, FOS or MOS oligosaccharides at concentrations of 50, 500 and 1000 µg/mL was added to the cells and the cells with their respected oligosaccharide treatments were incubated for two hours. For the control, only 0.25 mL of antibiotic-free RPMI medium was added to the cells prior to the two-hour incubation. After two hours, 0.25 mL of RPMI medium containing *S.* Enteritidis (approximately 5.0 × 10^5^ CFU/mL) was added and the cells were incubated at 37 °C for another 30 min to allow for *S.* Enteritidis colonization. After incubation, the supernatant from the cells were collected and stored in a −80 °C freezer for LDH analysis (0 h post-infection). The cells were then rinsed thrice with serum-free and antibiotic-free RPMI media. After rinsing, RPMI medium containing 100 μg/mL gentamicin (Nacalai Tesque, Kyoto, Japan) was added to eliminate extracellular *S.* Enteritidis. The cells were incubated with 100 μg/mL gentamicin for 4 h. After 4 h, the cells were washed twice with RPMI before RPMI medium containing 10 μg/mL gentamicin was added to prevent re-infection and growth of *S.* Enteritidis in the culture medium. At indicated time points (4, 9, 14 and 18 h post-infection), the cell culture supernatant was collected and the macrophages were lysed for 5 min with 0.5 mL of chilled PBS containing 0.1% Triton X-100. Once lysed, the lysate was serially diluted and plated onto BGA (Difco, Becton Dickinson Co., Franklin Lakes, NJ, USA). The agar plates were then incubated overnight at 37 °C in order to enumerate viable intracellular *S.* Enteritidis. For LDH analysis using the cell culture supernatant of U-937 macrophages, the methodology is identical to the one used for the LDH analysis of Caco-2 cells.

### 2.9. Statistical Analysis

Statistical analysis was carried out using one-way analysis of variance (ANOVA) followed by Tukey’s post-test for studies involving Caco-2 cells, while two-way ANOVA followed by Bonferroni post-test was used for studies involving U-937 on Prism Graph Pad software version 5.01(GraphPad, San Diego, CA, USA). The results shown are the mean values ± SEM for three biological replicates and a *p*-value of *p* < 0.05 was considered significant.

## 3. Results and Discussion

### 3.1. HPLC Profiles of PKC Oligosaccharides

**Figure 1:**
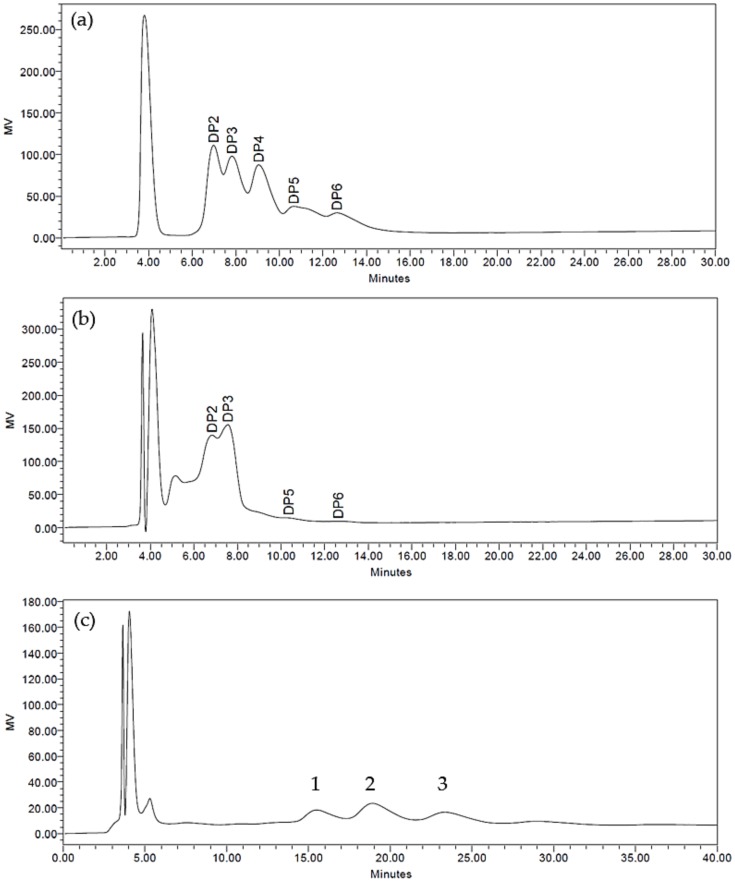
(**a**) High-Performance Liquid Chromatography (HPLC) profiles of oligosaccharide standards ranging from a degree of polymerization (DP) of two to six. These standards are mannobiose, mannotriose, mannotetraose, mannopentaose, and mannohexaose, respectively. (**b**) HPLC profiles of the Small oligosaccharide fraction (DP ≤ 6) showing peaks consisting of mainly DP2 and DP3. (**c**) HPLC profiles of the Big oligosaccharide fraction (appearing at 15 min and later) showing peaks consisting largely of DP greater than (DP > 6).

High-Performance Liquid Chromatography (HPLC) is a popular and robust analytical technique commonly used for the separation, identification and quantification of analytes in a mixture [31]. In this study, HPLC coupled with an aminopropyl column and a Refractive Index Detector (RID) was used to ensure that the pooled oligosaccharide fractions contained oligosaccharides of different compositions from one another. From the HPLC profiles (Figure 1), both the Big and Small oligosaccharide fractions were discrete from each other with the Small fraction consisting mostly of oligosaccharides with lower DP, with most of the peaks appearing before 8 min, coinciding with the retention times of DP2 (mannobiose) and DP3 (mannotriose) of the standards. The majority of the Big fraction was made up of oligosaccharides, with higher DP and a retention time above 15 min. The use of water as a solvent for the extraction of soluble oligosaccharides from PKC has been reported in several studies [27,32,33]. Of its sugar constituents, mannose was reported to be the predominant monosaccharide in PKC, with a mannose content ranging from 45.1% to 57.3% [27,32]. Other monosaccharides reported to be present are galactose, glucose, xylose, fructose, arabinose and rhamnose [27,32].

### 3.2. Molecular Weights of Oligosaccharides

While the HPLC analysis used in this study provided a tentative compositional profile of the Small and Big oligosaccharide fraction, LC-QTOF/MS was able to provide information concerning the relative formula mass of the oligosaccharides present and thus enable a more accurate determination of the DP of the oligosaccharides present in the Small, Big, MOS and FOS. From the results in Table 1, the Small and FOS oligosaccharides contained saccharides ranging from 1 DP (monosaccharide) to 6 DP (hexasaccharide). The Big oligosaccharide fraction contained saccharides ranging from 5 DP (pentasaccharide) to 10 DP (decasaccharide). The presence of heptasaccharide, octasaccharide and decasaccharide may correspond to the unknown peaks 1, 2 and 3 from Figure 1C, respectively, based on the molecular weight observed. These findings differ slightly from that of Jahromi et al. (2016) who reported the presence of oligosaccharides up to 8 DP in the aqueous PKC extract [27]. A probable explanation could be due to the longer run time employed in this study for the HPLC analysis as the peak for decasaccharides could only be detected after 23 min. For the commercial MOS, only the presence of monosaccharides could be detected. This could imply that the commercial MOS, which is obtained from yeast cell walls, may consist of polysaccharides that are larger than 10 DP or that its saccharides may exist as derivatives and are thus not recognized during the targeted LC-QTOF/MS analysis [34,35]. While the separation of the oligosaccharide standards were good and oligosaccharides of similar molecular weight in the samples could be identified through LC-QTOF/MS analysis, the separation of the monosaccharide standards could not be achieved with the isomers glucose, fructose, galactose and mannose and these monosaccharides appear as a single peak with an observed molecular weight of 179.1. The difference between the expected and observed molecular weights of the analytes is due to the deprotonation (removal of hydrogen (H^+^) ion) of the analyte when run in the negative mode [36].

### 3.3. Salmonella Enteritidis Adherence Inhibition to Caco-2 Cells

**Figure 2:**
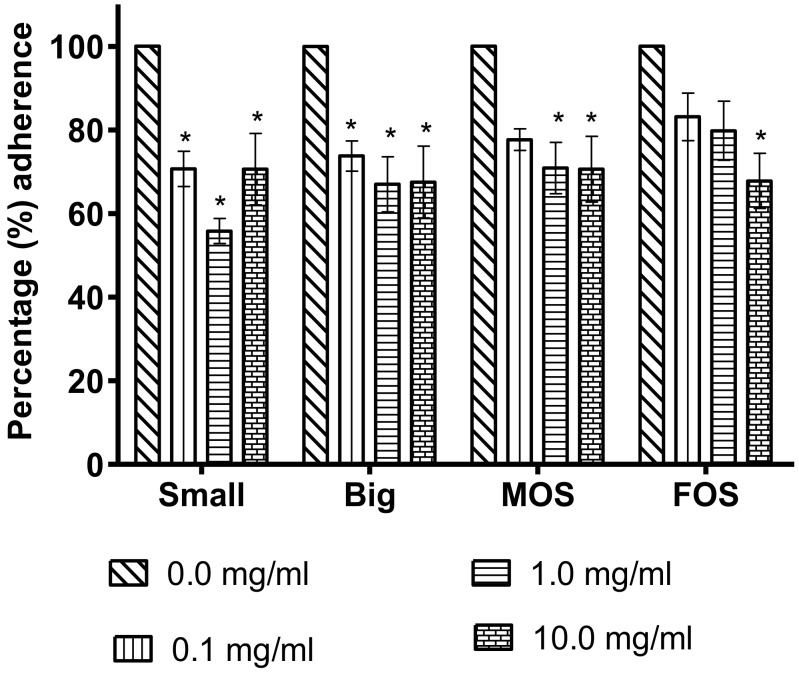
Effect of oligosaccharide treatment on the percentage inhibition of *Salmonella* Enteritidis adherence to Caco-2 cells as compared to the control. The values represent the mean ± SEM of three biological replicates. Significant values within the same row are represented by the asterisk symbol “*” = *p* < 0.05. Small = Palm kernel cake oligosaccharides with a degree of polymerization equal to or less than six (DP ≤ 6). Big = Palm kernel cake oligosaccharides with a degree of polymerization larger than six (DP > 6). MOS = mannanoligosaccharide. FOS = fructooligosaccharide.

The anti-adherence assay was carried out to evaluate the ability of the tested NDOs to reduce *S*. Enteritidis adherence to Caco-2 cells, as the adhesion of a pathogen to the host cell is essential for a successful infection to take place. The effects of the two PKC and the commercial oligosaccharides on *S.* Enteritidis adherence to Caco-2 cells are presented in Figure 2. Both the Big and Small PKC fractions were capable of inhibiting *S.* Enteritidis adherence at 0.1 mg/mL (*p* < 0.05), while MOS and FOS showed significant adherence inhibition only at higher concentrations, 1.0 mg/mL and 10.0 mg/mL, respectively, as compared to the untreated control. At 0.1 mg/mL concentration, the Small fraction showed a reduction (*p* < 0.05) of 29.3% on *S.* Enteritidis adherence as compared to the control and the reduction further increased to 44.2% at concentration of 1.0 mg/mL which is the highest among the four oligosaccharides suggesting that the Small DP PKC oligosaccharide has the highest capability in preventing *S.* Enteritidis attachment to the epithelial cells. The ability of oligosaccharides to reduce *S.* Enteritidis attachment to epithelial cells is important because upon entering the digestive tract, one of the first barriers faced by the pathogen is the mucosal lining, which provides a non-specific physical and chemical barrier against invading pathogens [3,37]. In order to overcome this barrier and infect the host, a sufficient number of pathogens must first bind to the epithelial cell [37]. To do this, pathogens possess adhesins which target and bind to specific receptors on the cellular surface [38]. *S.* Enteritidis have Type 1 fimbrial FimH adhesins which have a high affinity for mannose [39]. This mannose sensitivity enables mannose to play an important role as a receptor analog to *S.* Enteritidis and this in turn inhibits *S.* Enteritidis attachment and therefore limits the crucial step needed to a successful infection [38,39].

The Big fraction is slightly less efficient than its Small counterpart at reducing *S.* Enteritidis adherence with a maximum adherence inhibition of 33.0% at 1.0 mg/mL. According to Old (1972), of the six carbon atoms that make up the monosaccharide mannose, it is the hydroxyl group of C2, C3, C4, C5 and C6 that plays a role in binding to the FimH fimbrial protein. However, the formation of glycosidic bonds in oligosaccharides might reduce the number of hydroxyl groups that is able to interact with the fimbrial proteins of *S.* Enteritidis. Therefore, the longer the oligosaccharide chain, such as those that are found in the Big oligosaccharide fraction, the fewer the hydroyxl sites available for interaction when compared to the Small oligosaccharide fraction.

While the commercial MOS showed a reduction in *S.* Enteritidis adherence to the Caco-2 cells, it required a higher concentration (1.0 mg/mL) to achieve a significant reduction as compared to the Big and Small oligosaccharide fractions. A plausible explanation for this may be due to the differences in their compositions. The MOS used in this study is a commercial product which claimed to contain 99% mannanoligosaccharides but its degree of polymerization was not specified [34]. LC-QTOF/MS could not detect the presence of saccharides between 2 and 10 DP in MOS confirming that its oligosaccharide composition is different from that of the Big and Small oligosaccharide fraction.

Likewise, the FOS required a higher concentration (10.0 mg/ mL) to achieve comparable adherence inhibition to that of other oligosaccharides. The moderately inhibitive effect of FOS is in agreement with the findings of Old (1972), which reported that D-mannose was capable of inhibiting *Salmonella* Typhimurium fimbria1 haemagglutination at 0.02% *w*/*v* as compared to D-fructose which required up to 0.2% *w*/*v* because D-fructose may exert its inhibitive properties by attaching itself onto fimbrial sites which are not mannose sensitive [40].

The concentration of oligosaccharides used for the adherence inhibition assay is within reported dietary supplementations. For example, FOS, which is a commonly used food ingredient, has been awarded the Generally Recognized as Safe (GRAS) status and the Food and Drug Administration (FDA) reports that FOS up to 20 g/day is safe for the general population above one year of age [41]. While MOS does not have the GRAS status, animal trials with MOS supplementation at a dose of 1% (10 mg/mL) showed no adverse effect [42,43]. Likewise, no adverse effects were observed in in vivo trials when oligosaccharides from PKC at a concentration of 10 mg/mL were used as supplements [44,45].

### 3.4. Effects of Oligosaccharides on the Release of LDH in LPS-Induced Caco-2 Cells

Bacterial endotoxin, also known as lipopolysaccharide (LPS) is a strong inducer of proinflammatory response, which when generated in excess leads to the production of reactive oxygen species (ROS) and cellular damage [46,47]. The extent of LPS-induced cellular damage may be assessed by measuring the amount of LDH released from cells whose membrane integrity has been compromised [48]. Although LPS is a strong proinflammatory trigger and is major part of the outer membrane of Gram-negative bacteria, it is interesting to note that only highly virulent strains of *S.* Enteritidis appear capable of inducing significant LDH release in Caco-2 cells [49].

**Figure 3:**
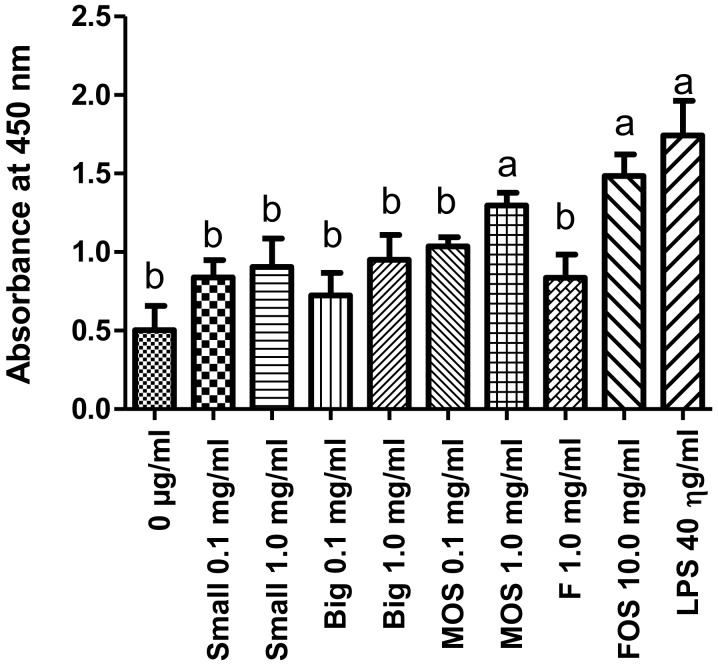
Effects of oligosaccharide treatment on lactate dehydrogenase (LDH) release in lipopolysaccharide (LPS)-induced Caco-2 cells. The negative control consists of untreated cells (0 µg/mL), while the positive control consists of only LPS at 40 ηg/ mL. Test parameters consisted of 40 ηg/mL LPS and oligosaccharides at indicated concentrations. The values represent the mean ± SEM of three biological replicates. Treatment groups containing the letter “a” are not statistically significant from one another at *p* < 0.05 but are statistically different at *p* < 0.05 from treatment groups containing the letter “b” and vice versa. Small = Palm kernel cake oligosaccharides with a degree of polymerization equal to or less than six (DP ≤ 6). Big = Palm kernel cake oligosaccharides with a degree of polymerization larger than six (DP > 6). MOS = mannanoligosaccharide. FOS = fructooligosaccharide.

From the results in Figure 3 (absorbance values available in Supplementary Table S1), Caco-2 cells that were treated with LPS alone showed a significantly higher LDH release and hence cellular damage when compared to the untreated control. However, when LPS and oligosaccharides were given together (as shown in Figure 3), the effects of LPS on LDH release was reduced significantly by Small at 0.1 and 1.0 mg/mL; Big at 0.1 and 1.0 mg/mL; MOS at 0.1 mg/mL; and FOS at 1.0 mg/mL. The observed effects might be attributed to the purported antioxidant activities of oligosaccharides, which reduces the oxidative stress caused by LPS [50,51,52]. While both MOS at 1.0 mg/mL and FOS at 10 mg/mL were able to significantly reduce *S.* Enteritidis adherence to Caco-2 cells, these oligosaccharide concentrations were unsuitable for the prevention of cellular damage caused by LPS. 

### 3.5. Intracellular Clearance of Salmonella Enteritidis in U-937 Macrophages

**Figure 4:**
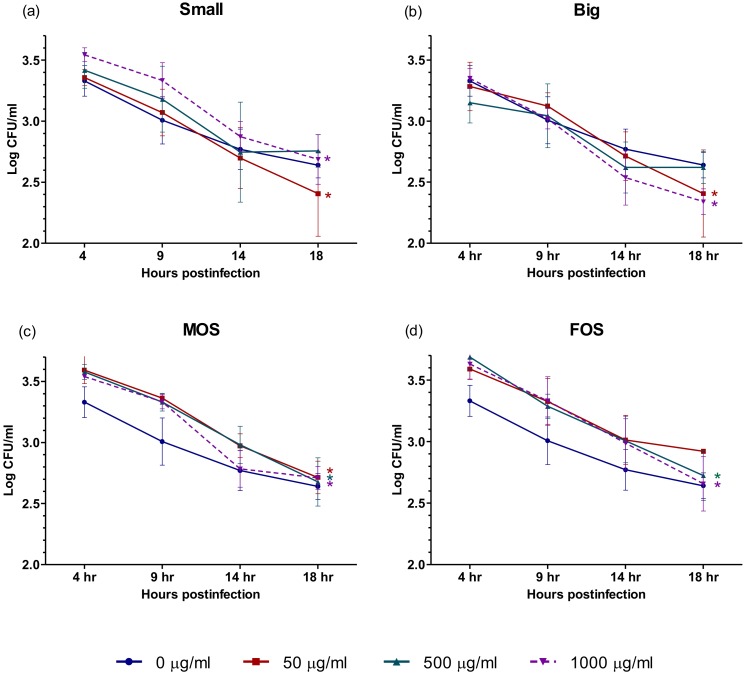
Effect of (**a**) Small; (**b**) Big; (**c**) MOS; and (**d**) FOS on the rate of intracellular *S.* Enteritidis clearance in U-937 macrophages. The values represent the mean ± SEM of three biological replicates. Means containing the asterisk symbol “*” within a treatment group are considered significantly different at *p* < 0.05 when compared to the log CFU/ mL at 4 h post-infection. The different colored asterisk symbol “*” corresponds to the different oligosaccharide concentration used. Small = Palm kernel cake oligosaccharides with a degree of polymerization equal to or less than six (DP ≤ 6). Big = Palm kernel cake oligosaccharides with a degree of polymerization larger than six (DP > 6). MOS = mannanoligosaccharide. FOS = fructooligosaccharide.

Macrophages are one of the key players in the innate immune system and function not only as the second line of defense against pathogens after the epithelial barrier is breached but also in regulating inflammation and wound healing [53]. In order to defend the host against invading pathogens, macrophages possess a broad range of receptors and are able to differentiate self from non-self through the recognition of pathogen-associated molecular patterns [3]. Several of these receptors are known to react to oligosaccharides [54,55]. Studies have also shown that the activation of macrophages may lead to the faster clearance of *Salmonella* [18,56]. The rapid clearance of *Salmonella* is desired to avoid the establishment of a persistent infection and the complications that come with it. Results (Figure 4, Supplementary Table S2) indicated that all four oligosaccharides tested were capable of causing significant reduction of *S*. Enteritidis over time. At 50 µg/mL, Small, Big and MOS showed a marked reduction of *S*. Enteritidis at 18 h when compared to their initial 4 h time point. For FOS, 500 µg/mL was required to achieve a marked decrease in intracellular *S*. Enteritidis at 18 h. While also showing a decreasing trend, the decrease in intracellular *S*. Enteritidis in the control (0 µg/mL) over time was not significant.

### 3.6. LDH Measurement and Correlation between LDH Levels and Intracellular Salmonella Enteritidis Clearance in U-937 When Treated with Non-Digestible Oligosaccharides

**Figure 5:**
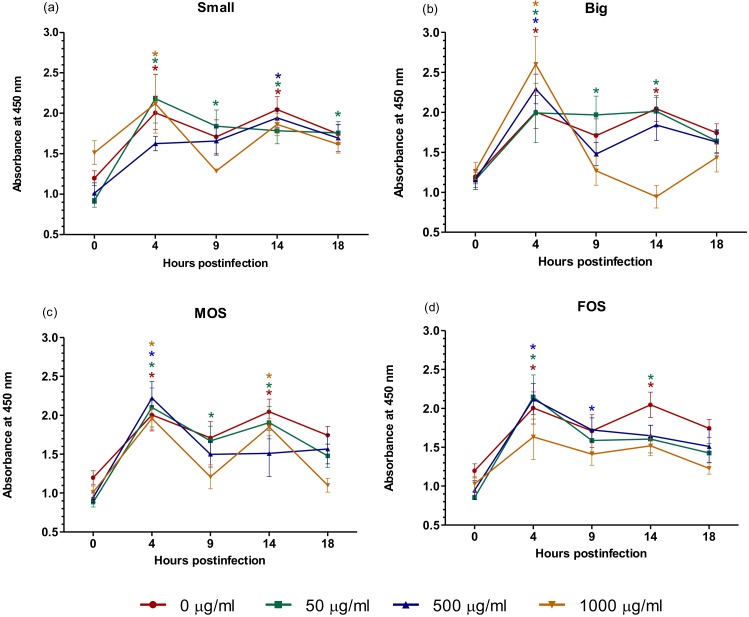
Effect of (**a**) Small; (**b**) Big; (**c**) MOS; and (**d**) FOS on LDH released by U-937 macrophages infected with *S*. Enteritidis. The values represent the mean ± SEM of three biological replicates. Means containing the asterisk symbol “*” within a treatment group are considered significantly different at *p* < 0.05 when compared to the absorbance measurement at 0 h post-infection. The different colored asterisk symbol “*” corresponds to the different oligosaccharide concentration used. Small = Palm kernel cake oligosaccharides with a degree of polymerization equal to or less than six (DP ≤ 6). Big = Palm kernel cake oligosaccharides with a degree of polymerization larger than six (DP > 6). MOS = mannanoligosaccharide. FOS = fructooligosaccharide.

In order to determine whether the increased rate of *S*. Enteritidis clearance observed in NDO-treated macrophages would correlate with lower LDH levels, supernatants from the macrophage cultures were collected for analysis, The effect of oligosaccharides on the release of LDH when *S.* Enteritidis is introduced to U-937 macrophages is shown in Figure 5 (Supplementary Table S3 and S4). With the exception of Small at 500 µg/mL and FOS at 1000 µg/mL, a sharp increase (*p* < 0.05) in LDH was observed from 0 h (initial) to 4 h post-infection for the control (0 µg/mL), Small, Big, MOS and FOS. The initial sharp rise in LDH levels is expected as it has been reported that macrophages release LDH during and immediately after phagocytosis [57]. However, the release of LDH from activated macrophages is not linked solely to cell death as the LDH levels would gradually decrease over time [57]. A second spike in LDH levels was observed in all treatment groups at 14 h post-infection. This increase in LDH levels could be linked to a delayed cell death response exhibited by macrophages infected with *S.* Enteritidis [58,59]. Although the graphs in Figure 5 shows two peaks corresponding to increased levels of LDH at 4 and 14 h post-infection, the mechanisms underlying the observed results appear to be rather different. According to a study published by Santos et al. (2001) and Valle and Guiney (2005), the initial and early cell death, as evidenced by an increase in LDH levels at 4 h post-infection is linked to *Salmonella* pathogenicity island 1 (SPI-1) while the second LDH increase at 14 h post-infection is associated with *Salmonella* pathogenicity island 2 (SPI-2). The delayed cell death response in U-937 macrophages treated with 1000 µg/mL Big appears to be delayed even further with the second increase in LDH appearing at 18 h post-infection. Of interest is also the trend exhibited by Small 50 µg/mL, Big 50 µg/mL, MOS 500 µg/mL, FOS 50 µg/mL and FOS 500 µg/mL. This is because U-937 macrophages treated with the aforementioned oligosaccharide concentrations do not exhibit a rise in LDH levels at 14 h post-infection. It would be interesting to find out in future studies whether the NDOs used at those concentrations had antiapoptotic effects on macrophage cells or whether NDOs at those concentrations were inhibiting the expression of SPI-2 genes in *S.* Enteritidis.

Table 2 shows the degree of the relationship between the numbers of intracellular *S.* Enteritidis in U-937 macrophages and the macrophages’ corresponding LDH levels from 4 to 18 h post-infection. A strong correlation between variables has a correlation coefficient (r^2^) value between 0.70 and 1.00, a moderate correlation ranges from 0.30 to 0.69, and a weak correlation between variables has a r^2^ value of less than 0.3 [60]. Of the four oligosaccharides tested, FOS at 500 µg/mL showed the strongest correlation between decreasing intracellular *S.* Enteritidis and LDH levels, with an r^2^ value of 0.93. The Small oligosaccharide fraction also showed a strong correlation between decreasing *S.* Enteritidis and LDH values, with an r^2^ value of 0.74 when given at a dose of 50 µg/mL. Both the Big and MOS on the other hand showed a moderate correlation at 50 µg/mL with an r^2^ value of 0.57 and 0.51, respectively. As for the untreated control, only a weak correlation (r^2^ = 0.10) was observed between the numbers of intracellular and the corresponding LDH levels. It is possible that the slower rate of clearance exhibited by the untreated macrophages may have led to higher cellular damage and delayed cell death [58,61,62].

In summary, the results indicate that both the Small and Big oligosaccharide fractions from PKC compare favorably with, if they are not better than, the commercial prebiotics MOS and FOS, with the Small performing better than the Big at reducing *S.* Enteritidis adherence to Caco-2 cells and improving *S.* Enteritidis clearance in U-937 macrophages. These findings have, to our knowledge, not been reported. However, the utilization of oligosaccharides from PKC as a commercially sustainable and alternative source of NDOs would require further experiments involving in vivo studies in order to ensure that the overall effect of NDOs from PKC on a living host is a net positive.

## Figures and Tables

**Table 1:** Molecular weight of monosaccharides and oligosaccharides found in Small, Big, MOS and FOS.

Sugar	Molecular Formula	Expected Molecular Weight	Observed Molecular Weight			
			Small	Big	MOS	FOS
Monosaccharide	C_6_H_12_O_6_	180.2	179.1	-	-	179.1
Disaccharide	C_12_H_22_O_11_	342.3	341.1	-	-	341.1
Trisaccharide	C_18_H_32_O_16_	504.4	503.1	-	-	503.1
Tetrasaccharide	C_24_H_42_O_21_	666.6	666.2	-	-	666.2
Pentasaccharide	C_30_H_52_O_26_	828.7	828.3	828.3	-	828.3
Hexasaccharide	C_36_H_62_O_31_	990.9	990.3	990.3	-	990.3
Heptasaccharide	C_42_H_72_O_36_	1153	-	1152.4	-	-
Octasaccharide	C_48_H_82_O_41_	1315.2	-	1314.4	-	-
Nonasaccharide	C_54_H_92_O_46_	1477.3	-	-	-	-
Decasaccharide	C_60_H_102_O_51_	1639.5	-	1638.5	-	-

**Table 2:** Correlation between intracellular *Salmonella* Enteritidis in U-937 macrophages and LDH released. The values represent the mean ± SEM of three biological replicates.

Oligosaccharides	Correlation Coefficient (r^2^) Values			
	0 µg/ mL	50 µg/ mL	500 µg/ mL	1000 µg/ mL
Control	0.10	-	-	-
Small	-	0.74	0.50	0.04
Big	-	0.57	0.15	0.54
MOS	-	0.51	0.46	0.11
FOS	-	0.78	0.93	0.67

Small = Palm kernel cake oligosaccharides with a degree of polymerization equal to or less than six (DP ≤ 6). Big = Palm kernel cake oligosaccharides with a degree of polymerization larger than six (DP > 6). MOS = mannanoligosaccharide. FOS = fructooligosaccharide.

## Supplementary Materials

The following are available online at https://www.mdpi.com/2076-2607/8/2/255/s1. Supplementary materials have been uploaded along with the manuscript. Table S1: Absorbance value for the effects of oligosaccharide treatment on LDH release in LPS-induced Caco-2 cells; Table S2: Log CFU/mL values for the effect of oligosaccharide on the rate of intracellular *S.* Enteritidis clearance in U-937 macrophages; Table S3: Absorbance value from 0 to 9 h post-infection for the effect of oligosaccharides on LDH released by U-937 macrophages infected with *S.* Enteritidis; Table S4: Absorbance value from 14 to 18 h post-infection for the effect of oligosaccharides on LDH released by U-937 macrophages infected with *S.* Enteritidis.
