# Supplementary material for: Oligosaccharides from Palm Kernel Cake Enhances Adherence Inhibition and Intracellular Clearance of Salmonella enterica Serovar Enteritidis In Vitro
Source: Microorganisms. 2020 Feb 14;8(2):255. doi: 10.3390/microorganisms8020255 (PMC7074813; doi:10.3390/microorganisms8020255)
Supplement: Supplementary file 1 [file microorganisms-08-00255-s001.pdf]

Supplementary Material

Table S1. Absorbance value for the effects of oligosaccharide treatment on LDH release in LPS induced Caco-2 cells. Each independent assay consists of cell culture supernatants pooled from three different wells of a 24-well plate and each pool has two technical replicates for the microplate readout.

	Oligosaccharide treatment (mg/ml)									
	**Control**	**Small**		**Big**		**MOS**		**FOS**		**LPS**
	0	0.1	1.0	0.1	1.0	0.1	1.0	1.0	10.0	4x 10^{-5}
Assay 1	0.169	1.134	1.139	0.774	0.503	0.976	1.617	0.305	1.668	2.217
	0.415	0.418	1.038	0.671	0.551	1.056	1.441	0.458	1.056	2.217
Assay 2	0.651	0.803	0.204	1.049	0.954	1.137	1.144	1.11	1.75	2.217
	1.202	0.69	0.536	1.162	0.944	1.258	1.313	1.011	1.901	1.526
Assay 3	0.341	0.87	1.147	0.206	1.469	0.87	1.134	1.181	1.354	1.243
	0.237	1.113	1.369	0.48	1.288	0.925	1.132	0.949	1.171	1.037

Table S2. Log CFU/ml values for the effect of oligosaccharide on the rate of intracellular *S. enteritidis* clearance in U-937 macrophages. Each independent assay represents the mean value of intracellular *S. entiritidis* obtained from three different wells of a 24-well plate.

Oligosaccharide	Hours (hr) post infection											
	4			9			14			18		
0 µg/ml	3.23	3.58	3.18	2.91	3.38	2.73	2.78	3.05	2.48	2.66	2.81	2.45
Small 50 µg/ml	3.29	3.49	3.29	3	3.43	2.78	3	2.89	2.2	2.65	2.85	1.72
Small 500 µg/ml	3.28	3.71	3.26	3.08	3.69	2.77	3.03	3.27	1.94	2.82	2.95	2.5
Small 1000 µg/ml	3.61	3.59	3.43	3.23	3.62	3.15	3.06	2.92	2.64	2.77	2.99	2.3
Big 50 µg/ml	3.01	3.67	3.17	3.02	3.34	3.01	2.71	3.06	2.37	2.47	2.99	1.76
Big 500 µg/ml	3	3.48	2.97	2.94	3.54	2.65	2.64	2.97	2.25	2.71	2.79	2.36
Big 1000 µg/ml	3.28	3.51	3.26	3.03	3.16	2.87	2.8	2.72	2.09	2.23	2.55	2.24
MOS 50 µg/ml	3.37	3.72	3.69	3.4	3.4	3.29	3.04	3.1	2.78	2.72	2.94	2.48
MOS 500 µg/ml	3.48	3.56	3.69	3.45	3.34	3.2	3.22	3.02	2.7	2.71	3	2.32
MOS 1000 µg/ml	3.55	3.52	3.55	3.22	3.41	3.37	2.88	2.98	2.49	2.67	2.89	2.57
FOS 50 µg/ml	3.43	3.7	3.64	3.03	3.67	3.28	3.11	3.3	2.63	2.94	2.93	2.89
FOS 500 µg/ml	3.57	3.88	3.61	3.21	3.48	3.17	3.07	3.28	2.67	2.9	2.95	2.32
FOS 1000 µg/ml	3.45	3.87	3.57	3.19	3.72	3.08	3.02	3.35	2.59	2.82	2.93	2.22

Table S3. Absorbance value from 0 to 9 hours post infection for the effect of oligosaccharides on LDH released by U-937 macrophages infected with *S. enteritidis*. Each independent assay consists of cell culture supernatants pooled from three different wells of a 24-well plate and each pool has two technical replicates for the microplate readout.

Oligosaccharide	Hours (hr) post infection																	
	0						4						9					
	Assay 1		Assay 2		Assay 3		Assay 1		Assay 2		Assay 3		Assay 1		Assay 2		Assay 3	
0 µg/ml	1.066	1.021	1.055	1.073	1.47	1.495	1.472	1.285	2.26	2.133	2.38	2.501	1.082	1.045	2.178	2.122	2.025	1.797
Small 50 µg/ml	0.73	0.617	1.028	0.931	1.107	1.111	1.372	1.459	2.378	2.169	3.389	2.311	1.275	1.206	2.419	2.205	1.994	1.931
Small 500 µg/ml	0.82	0.756	1.39	1.337	0.665	1.106	1.759	1.631	1.322	1.418	1.912	1.707	1.223	1.194	1.699	1.527	2.186	2.114

Small 1000 μg/ml	1.132	1.142	1.481	1.442	1.939	1.94	2.787	0.513	1.637	2.734	2.593	2.445	1.351	1.175	1.345	1.293	1.288	1.263
Big 50 μg/ml	0.841	0.867	1.246	1.328	1.318	1.333	1.513	1.509	3.163	3.165	1.346	1.26	2.29	0.894	2.084	1.79	2.436	2.32
Big 500 μg/ml	0.864	0.82	1.155	1.104	1.49	1.509	1.899	1.927	3.079	2.555	2.052	2.241	1.09	0.979	1.793	1.748	1.688	1.575
Big 1000 μg/ml	0.945	0.907	1.399	1.412	1.487	1.447	1.59	1.53	3.5	3.279	3.054	2.656	0.774	0.739	1.317	1.307	1.682	1.788
MOS 50 μg/ml	0.705	0.745	0.828	0.898	1.052	1.07	1.343	1.331	2.304	2.297	2.764	2.56	1.144	1.216	1.595	1.665	2.174	2.233
MOS 500 μg/ml	0.73	0.816	0.998	0.938	1.057	1.115	1.56	1.571	2.777	2.56	2.456	2.398	1.318	1.379	1.047	1.27	1.968	2.002
MOS 1000 μg/ml	0.784	0.742	1.105	1.11	1.19	1.137	1.532	1.557	2.238	2.28	1.992	2.147	0.923	1.034	0.944	0.967	1.702	1.68
FOS 50 μg/ml	0.756	0.811	0.818	0.902	0.923	0.916	1.315	1.259	2.756	2.833	2.347	2.366	1.114	1.137	1.742	1.807	1.828	1.897
FOS 500 μg/ml	0.588	0.684	0.873	0.971	1.269	1.304	1.497	1.496	2.37	2.474	2.427	2.447	1.328	1.393	1.641	1.607	2.278	2.087
FOS 1000 μg/ml	1.195	1.289	0.712	0.752	1.098	1.131	1.448	0.643	1.448	1.341	2.441	2.467	0.967	1.008	1.454	1.513	1.759	1.771

Table S4. Absorbance value from 14 to 18 hours post infection for the effect of oligosaccharides on LDH released by U-937 macrophages infected with *S. enteritidis*. Each independent assay consists of cell culture supernatants pooled from three different wells of a 24-well plate and each pool has two technical replicates for the microplate readout.

Oligosaccharide	Hours (hr) post infection											
	14						18					
	Assay 1		Assay 2		Assay 3		Assay 1		Assay 2		Assay 3	
0 μg/ml	1.539	1.549	2.118	2.336	2.317	2.41	1.531	1.33	1.976	1.674	2.021	1.922
Small 50 μg/ml	1.347	1.374	1.721	1.782	2.207	2.259	1.375	1.245	2.014	1.976	1.992	1.931
Small 500 μg/ml	1.717	1.736	1.847	1.749	2.33	2.278	1.227	1.134	1.867	1.803	2.08	2.041
Small 1000 μg/ml	1.454	1.456	1.954	1.916	2.076	2.314	1.48	1.172	1.695	1.606	1.953	1.799
Big 50 μg/ml	1.43	1.511	2.365	2.191	2.297	2.277	1.232	1.175	1.692	1.946	1.935	1.864
Big 500 μg/ml	1.507	1.444	1.57	1.635	2.429	2.461	1.44	1.121	1.586	1.578	2.079	1.969
Big 1000 μg/ml	0.881	0.921	1.276	1.4	0.484	0.7	1.044	1.47	2.233	1.554	1.123	1.179
MOS 50 μg/ml	1.277	1.319	1.95	2.101	2.211	2.57	1.1	1.174	1.297	1.431	1.978	1.89
MOS 500 μg/ml	1.651	1.699	1.975	1.85	0.035	1.852	0.953	1.001	1.765	1.793	1.894	1.99
MOS 1000 μg/ml	1.529	1.525	1.9	1.857	2.126	2.154	1.313	1.276	1.051	0.711	1.093	1.157
FOS 50 μg/ml	1.148	1.243	1.377	1.598	2.152	2.104	1.05	1.153	1.291	1.701	1.666	1.698

FOS 500 µg/ml	1.243	1.417	1.501	1.649	2.014	2.065	0.806	0.901	1.727	1.756	1.912	1.96
FOS 1000 µg/ml	1.19	1.221	1.461	1.504	2.014	1.726	1.015	1.048	1.214	1.224	1.407	1.461
